# Surgical Management of Facial Port-Wine Stain in Sturge Weber Syndrome

**DOI:** 10.7759/cureus.12637

**Published:** 2021-01-11

**Authors:** Bar Y Ainuz, Erin M Wolfe, S. Anthony Wolfe

**Affiliations:** 1 Plastic and Reconstructive Surgery, Florida International University, Herbert Wertheim College of Medicine, Miami, USA; 2 Plastic and Reconstructive Surgery, University of Miami Miller School of Medicine, Miami, USA; 3 Plastic and Reconstructive Surgery, Nicklaus Children's Hospital, Miami, USA

**Keywords:** sturge-weber syndrome, encephalotrigeminal angiomatosis, port wine stain, capillary malformation, mental health

## Abstract

Sturge Weber Syndrome is characterized by the classic triad of a facial port-wine stain, leptomeningeal angiomatosis, and glaucoma. The resultant facial vascular anomaly can lead to soft tissue and bone irregularities, causing psychosocial distress and mental health morbidity. When severe, patients can opt for multi-staged surgical intervention by reconstructive surgeons to restore normal symmetry and improve the aesthetic appearance of the face. This study reports a case of surgical correction for severe facial vascular malformation resulting in poor outcomes due to the associated mental comorbidities seen in Sturge Weber Syndrome. A 37-year-old male with previously diagnosed Sturge Weber Syndrome presented to the outpatient craniofacial clinic for surgical evaluation of a large facial tuberous hemangioma. The patient underwent multiple operations for facial reconstruction including a staged full-thickness skin graft, facial recontouring, and extracranial correction of vertical orbital dystopia. The case was complicated by the patient’s poorly controlled seizure disorder and psychosocial illness, resulting in self-mutilation of the repair and poor follow-up. Over the span of 10 years, the patient’s mental illness caused him to fail numerous attempts at facial restoration and ultimately led to a poor final result. The psychosocial distress seen in patients with Sturge Weber Syndrome can adversely affect surgical outcomes. Physicians should be mindful of the possible complications that can arise in these patients and have the clinical means to address them.

## Introduction

Sturge Weber Syndrome, also known as encephalotrigeminal angiomatosis, is a congenital neurocutaneous vascular malformation syndrome with an estimated incidence of one in every 20,000 to 50,000 live births [1-3]. The mode of inheritance is sporadic, and the pathophysiology is thought to be due to a somatic mosaic activating mutation in a copy of the GNAQ gene during embryogenic development [4,5]. Manifestations are variable but are usually characterized by involvement of the unilateral skin, brain, and eye resulting in a classic triad including ipsilateral facial capillary malformation known as port-wine stain, intracranial vascular malformation known as leptomeningeal angiomatosis, and ocular vascular malformations causing glaucoma [1,6-8]. The leptomeningeal involvement can result in severe seizure disorder and intellectual disability, while the facial port-wine stain extends across the trigeminal nerve distribution, affecting both bones and soft tissue and potentially causing severe facial disfigurement [9]. The management of the resulting facial disharmony is undertaken by reconstructive plastic surgeons and includes laser treatments and challenging surgical reconstruction of the soft tissue and bony malformation to restore symmetry [9-12].

## Case presentation

A 37-year-old male patient with previously diagnosed Sturge Weber Syndrome presented to the outpatient clinic inquiring about reconstructive treatment for a congenital vascular malformation that engulfed a considerable portion of his face. Past medical history was significant for a lifelong seizure disorder secondary to intracranial vascular angioma for which he was placed on several medications with poor symptom control. The patient was also medically treated for depression and anxiety and visited a psychologist regularly for the social strain his disease has caused him with family and peers. 

**Figure 1:**
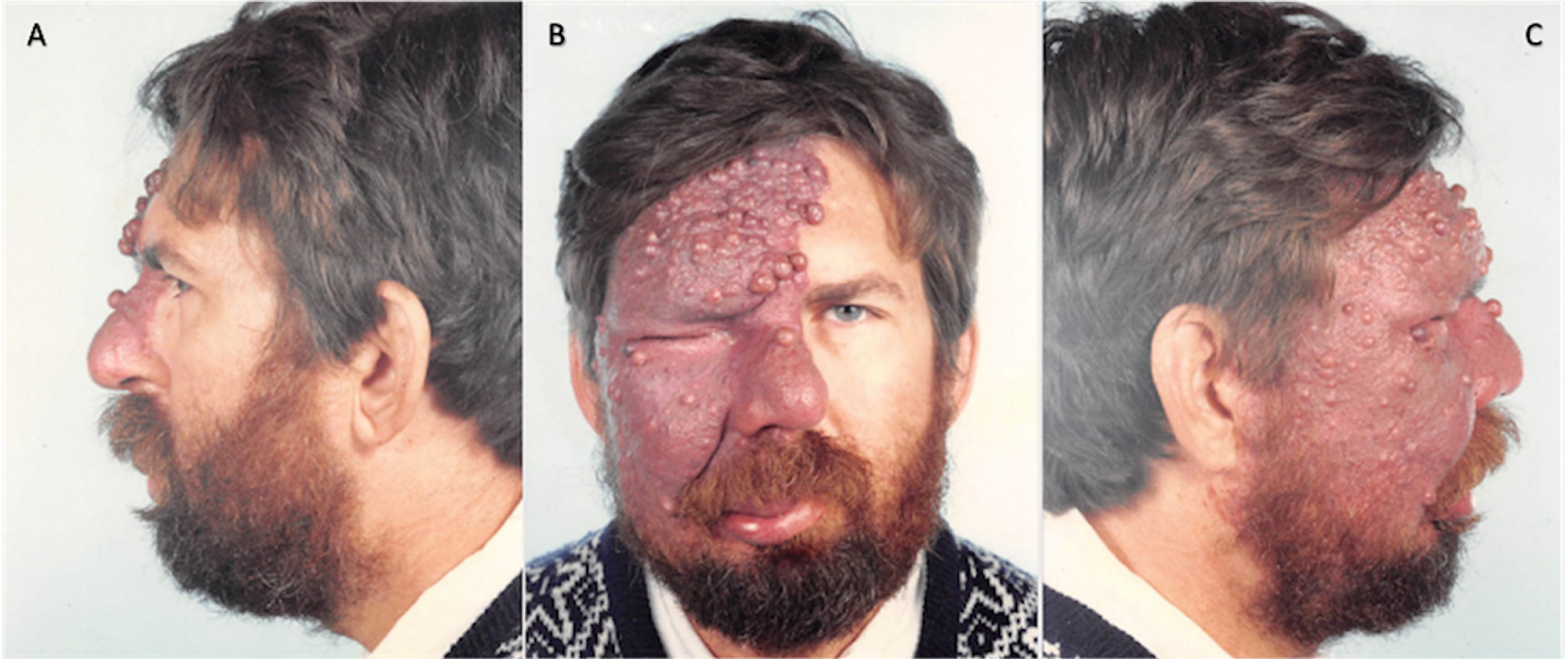
November 9, 1988. Preoperative images. A= Left profile, B= Frontal, C= Right profile

During the preoperative physical examination, the patient was noted to have a tuberous hemangioma of the right face and scalp with significant involvement in the frontal region (Figure 1). The patient had an intact vision at the time, was able to grow a beard over the affected side, and did not show any lesions on the oral examination. The patient was deemed a candidate for surgical intervention and scheduled to undergo excision of the facial vascular malformation with staged full-thickness skin graft reconstruction.

Procedure no. 1

**Figure 2:**
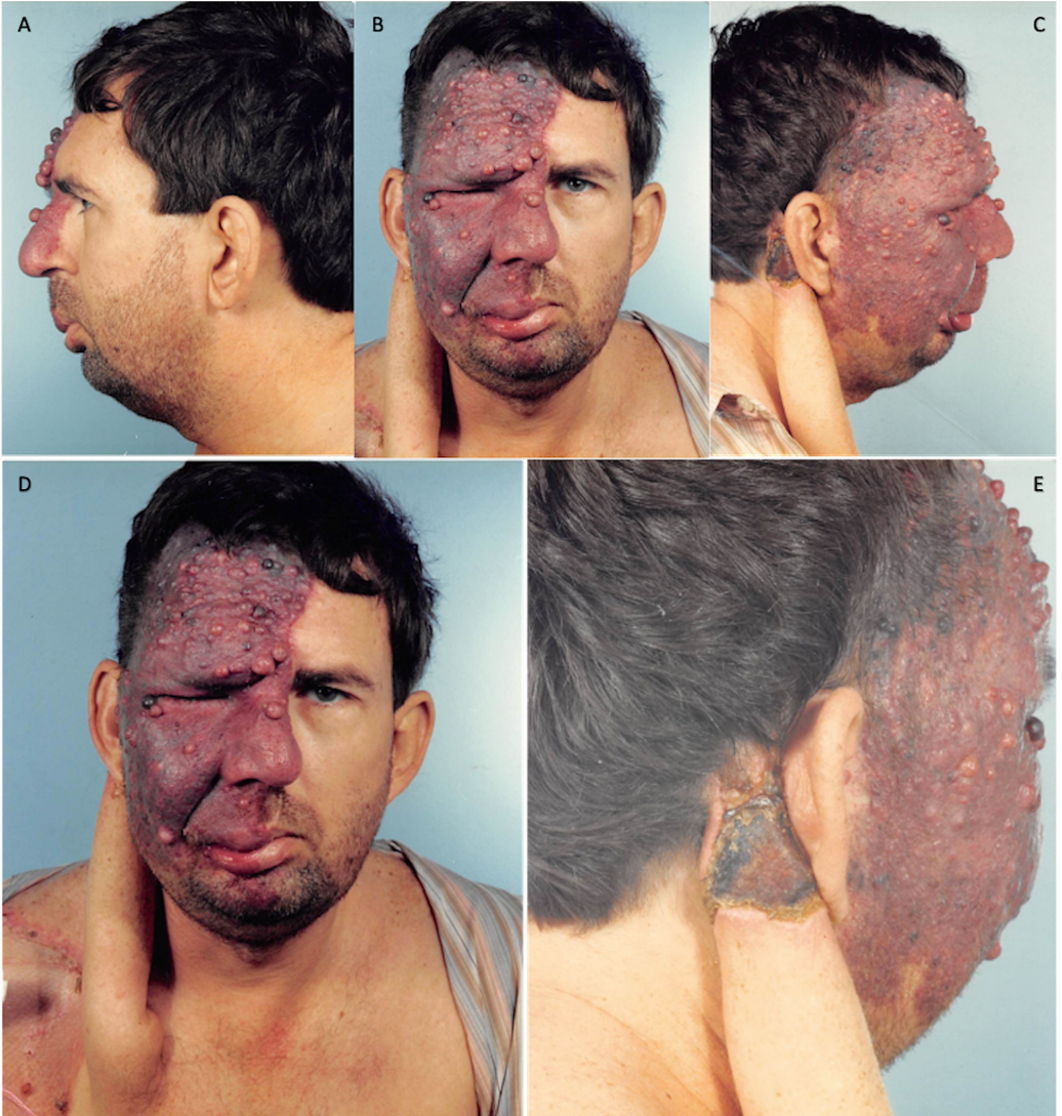
October 23, 1989. Postoperative procedure no. 1 images showing the raised deltopectoral flap with crusting and scaling at the donor insertion site. A= Left profile, B= Frontal, C= Right profile, D= Extended frontal, E= Posterior

A right deltopectoral flap was raised through the area of the perforators in the mid-chest, turned into a pedicle, and inserted into a donor site in the right postauricular area. In addition, a forehead flap was delayed, outlining the flap to involve the complete left forehead site. Postoperatively, the patient was assigned a home health nurse for daily wound care and dressing change. At one month follow up there was a dark area on the deltopectoral pedicle with crusting behind the ear indicating tissue damage, but the flap was otherwise healing well with no further deterioration and was deemed viable for future reconstruction (Figure 2).

Procedures no. 2 and 3

**Figure 3:**
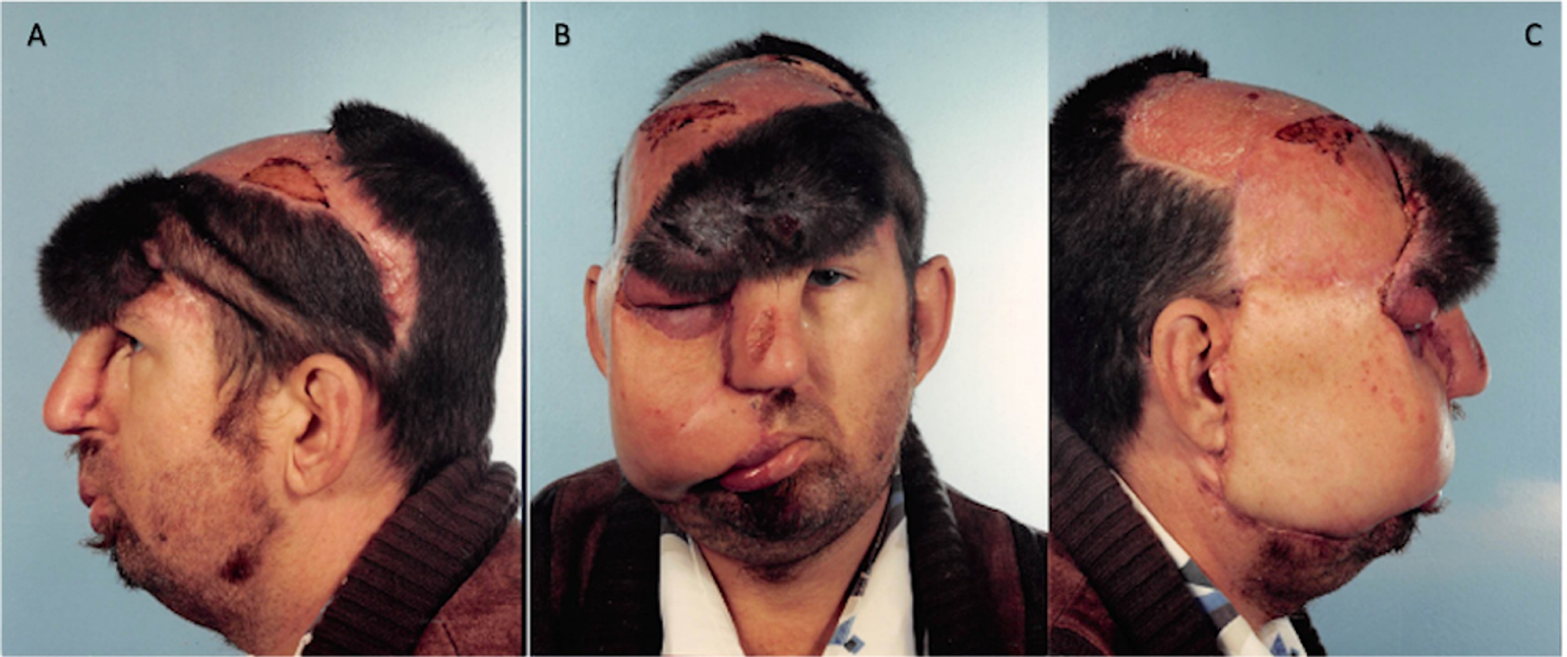
February 26, 1990. Postoperative procedures no. 2 and 3 images showing the deltopectoral flap covering the defect of the hemangioma excision site, with self-inflicted excoriation to the nose. A= Left profile, B= Frontal, C= Right profile

The hemangioma on the right forehead, cheek, and nose was excised. The tube pedicle was then divided from the chest, opened longitudinally, and tailored to the defect in the cheek and upper lip. The previously delayed left forehead flap was elevated from the forehead and rotated to the nasal area with the pedicle maintained in the left medial brow area. Two weeks later, the postauricular portion of the deltopectoral flap was divided and the flap was untubed. The remaining area of right cheek malformation and the right eyebrow was excised, and the flap was trimmed and tailored to fit the defect in the right cheek. Then, the scalp flap was placed into position to cover the defect over the right eyebrow. Postoperatively, the skin on the nose showed evidence of self-induced trauma indicated by a linear area of excoriation (Figure 3).

Procedure no. 4

**Figure 4:**
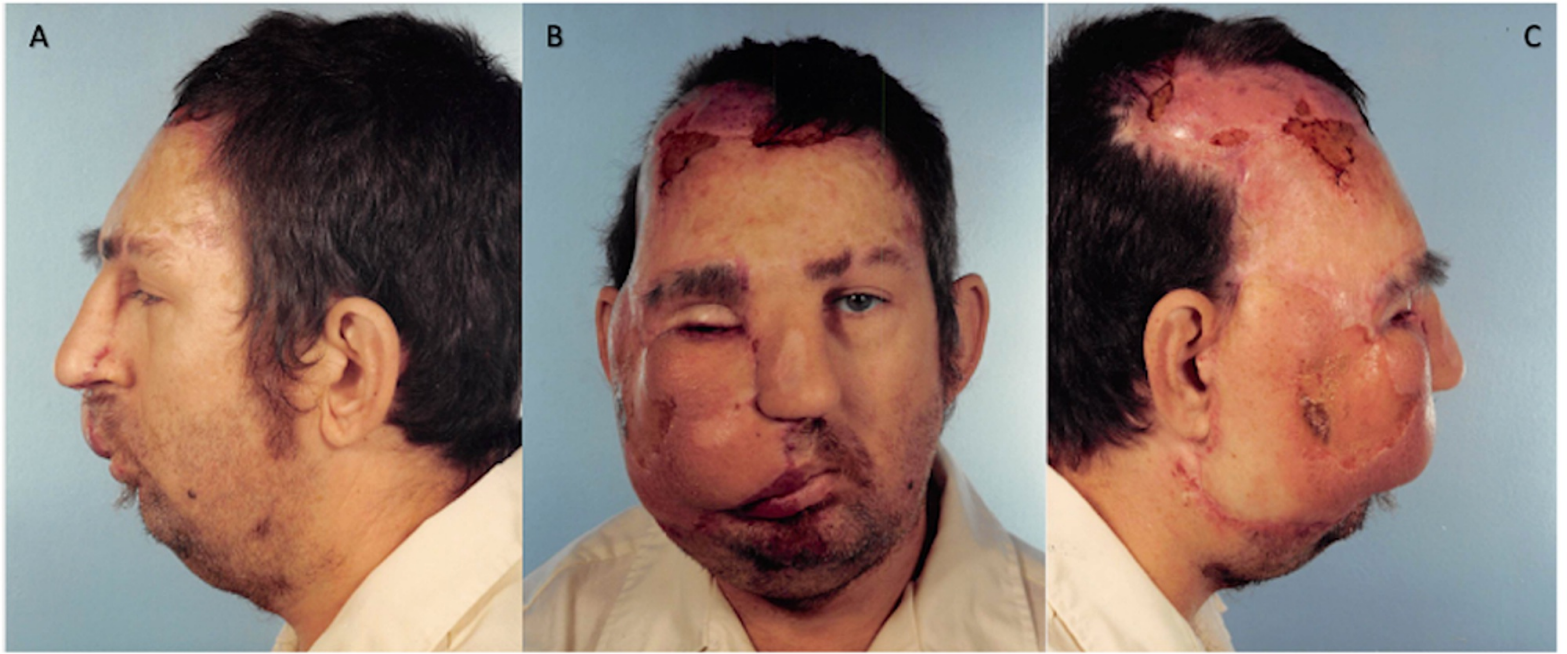
April 23, 1990. Postoperative procedure no. 4 images showing local wound breakdown over the scalp and forehead areas. A= Left profile, B= Frontal, C= Right profile

The patient underwent sculpting and recontouring of the flaps to create an anatomically appropriate eyebrow, upper eyelid, nasal bridge, and upper and lower vermillion borders on the right side of the face. Postoperatively, the patient continued to pick and scratch at large portions of his scalp flap and right cheek despite numerous warnings and safety measures including shielding the flaps with heavy dressing, prescribing antibiotics and anti-inflammatory medications to alleviate the associated pain and pruritus, and coordinating care with his psychologist to address these actions during therapy sessions. This resulted in local wound breakdown and necrosis which required repeated debridement and dressing during consequent visits (Figure 4).

Procedures no. 5 and 6

**Figure 5:**
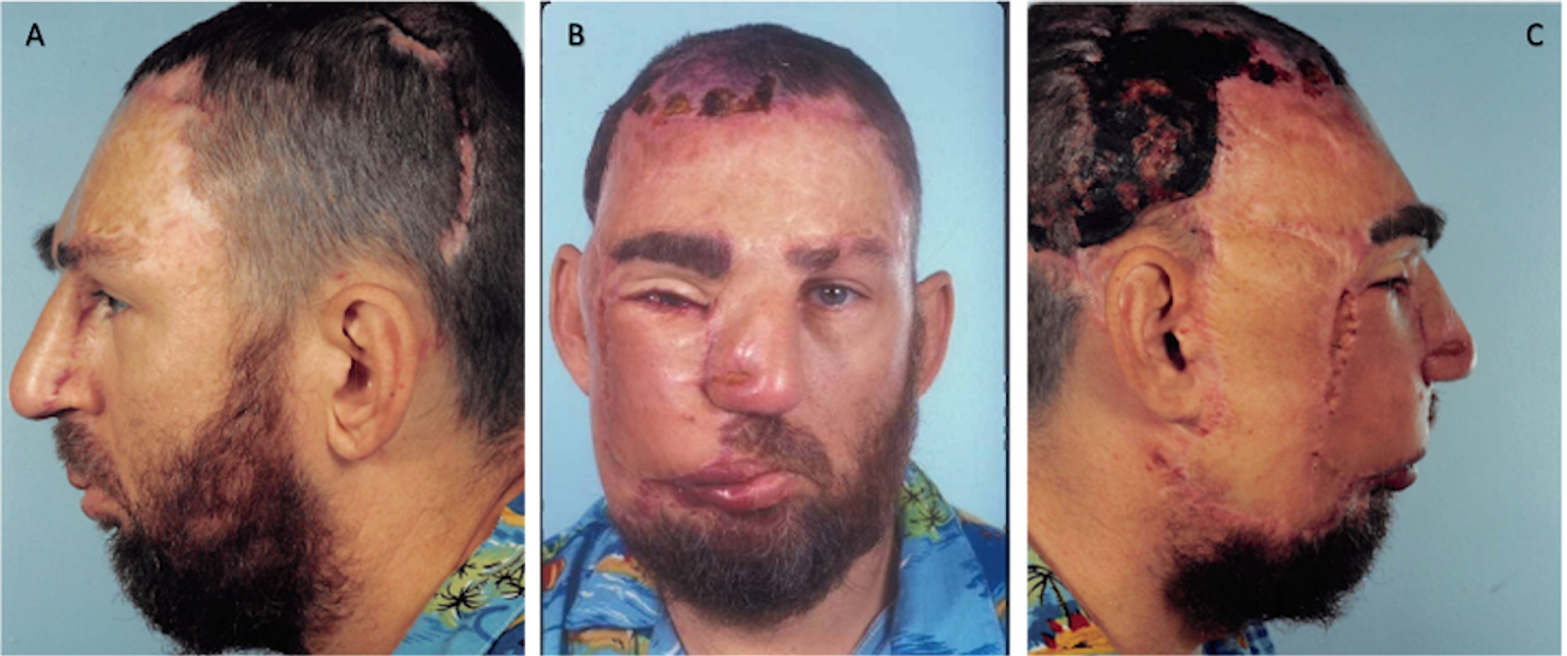
August 15, 1990. Postoperative procedures no. 5 and 6 images showing reconstitution of the frontal hairline and flap degeneration following failed tissue expander course. A= Left profile, B= Frontal, C= Right profile

**Figure 6:**
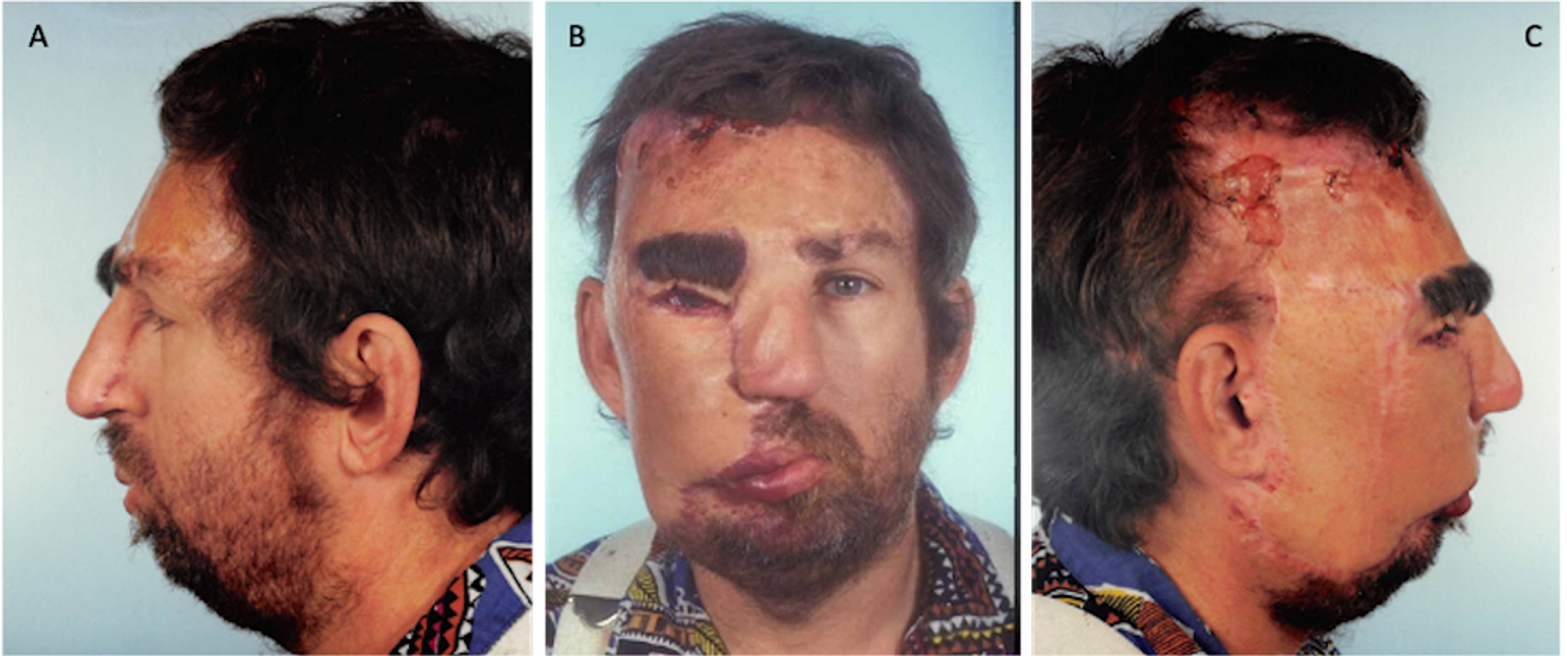
January 4, 1991. Images showing parietal and temporal scalp flap degeneration. A= Left profile, B= Frontal, C= Right profile

A tissue expander was inserted in the posterior scalp to promote the growth of additional skin to be used in the reconstitution of the anterior hairline. During the follow-up period, the patient missed several appointments for wound care and tissue expander fills. The tissue expander eventually became exposed, requiring prompt removal and further facial reconstruction (Figure 5). Postoperatively, the patient developed areas of necrosis and granulation tissue in the parietal and temporal regions as well as mummification of the scalp. Follow up remained poor and self-mutilation behaviors continued (Figure 6). Additionally, the patient began experiencing increased seizure activity with frequent hospital admissions which made it increasingly difficult to maintain a controlled environment for healing of the wounds. The overseeing neurologist managing his medication regimen described his seizures as both petit mal and tonic-clonic, with the latter type involving episodes of rapid left-sided contractions lasting up to two hours in duration. The prolonged and frequent tonic-clonic activity contributed to the patient's growing frustration and poor compliance with wound care. His psychologist voiced concerns regarding his internalized anger and tendency for impulsive behavior but advocated for continuing with surgical intervention as the patient was eager to improve his appearance. The focus shifted towards addressing the significant residual right vertical orbital dystopia and acquired scalp defects.

Procedure no. 7

**Figure 7:**
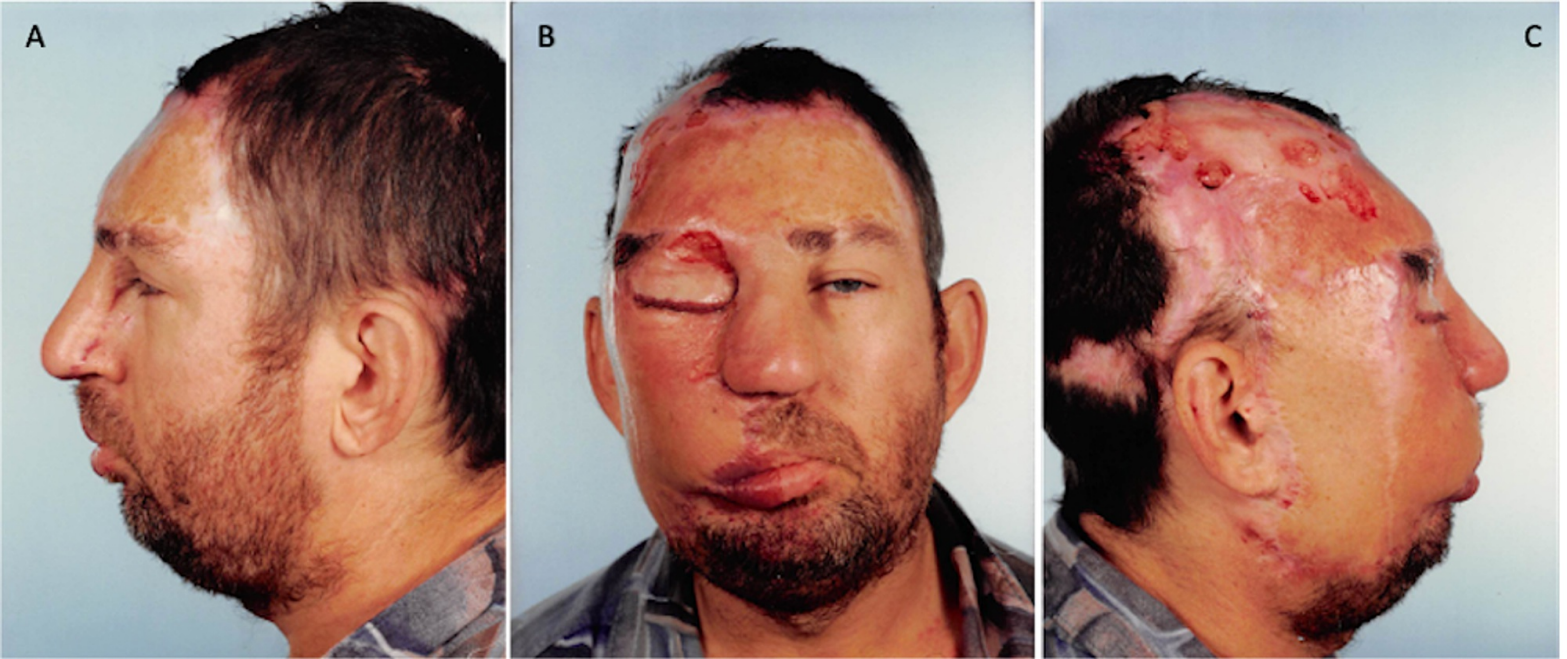
August 12, 1991. Postoperative procedure no. 7 images showing worsening flap degeneration secondary to repeated self-inflicted trauma. A= Left profile, B= Frontal, C= Right profile

The patient underwent correction of his right vertical orbital dystopia. Due to the large size of the frontal sinus discovered on imaging, an extracranial approach was chosen with orbital exploration, orbital unroofing into frontal and ethmoid sinuses, intraconal lipectomy, and medial canthopexy to establish symmetry with the contralateral unaffected side. Excision of multiple pyogenic granulomas in the right scalp was also performed followed by debulking and revision of the right facial flap, scalp flap advancement, and split-thickness skin graft for coverage of the remaining defects. Though the immediate postoperative period was well-tolerated, the patient continued to exhibit scratching/picking behavior of his facial wounds, causing severe tissue degeneration. The patient was seen at the office multiple times per week but attempts to prevent further self-injury were unsuccessful (Figure 7).

Procedure no. 8

**Figure 8:**
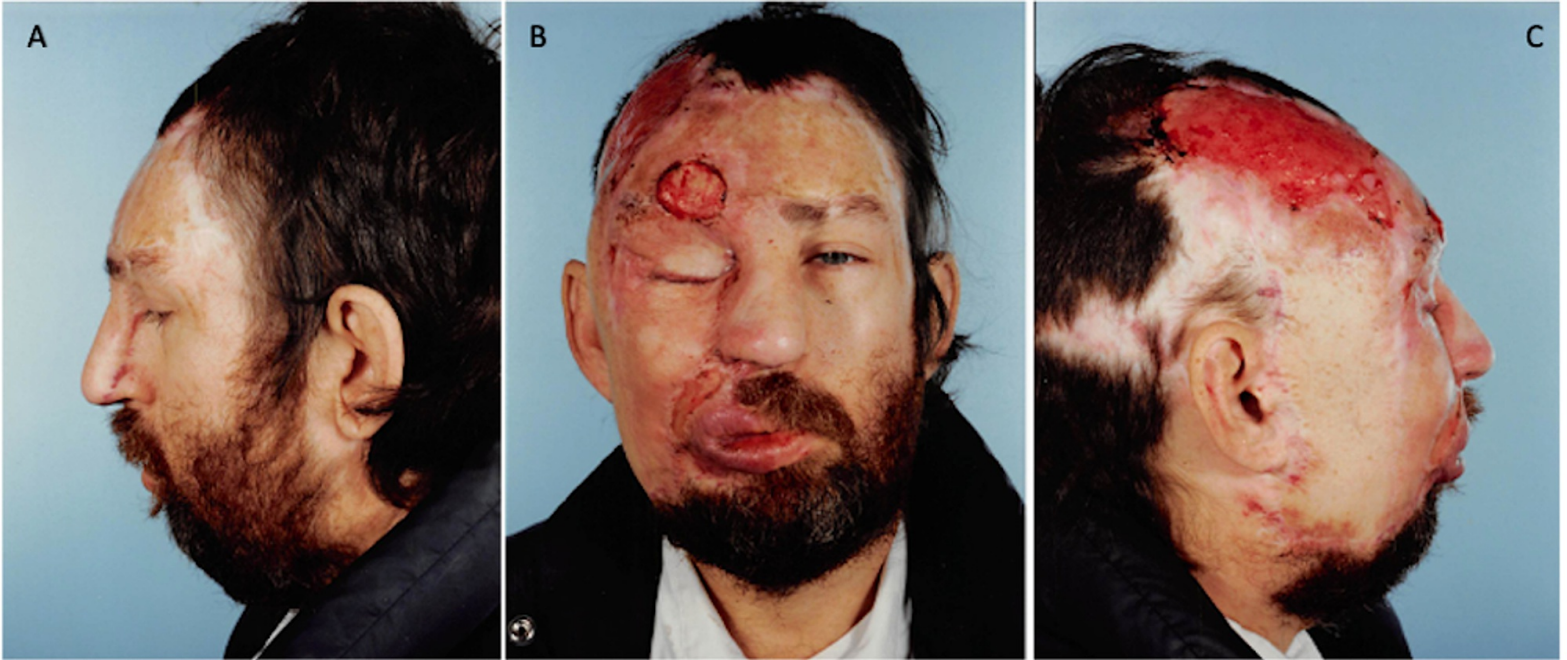
November 16, 1992. Postoperative procedure no. 8 images showing complete loss of the skin graft on the scalp and forehead, and partial loss in the right eyebrow and nasolabial fold following self-removal. A= Left profile, B= Frontal, C= Right profile

**Figure 9:**
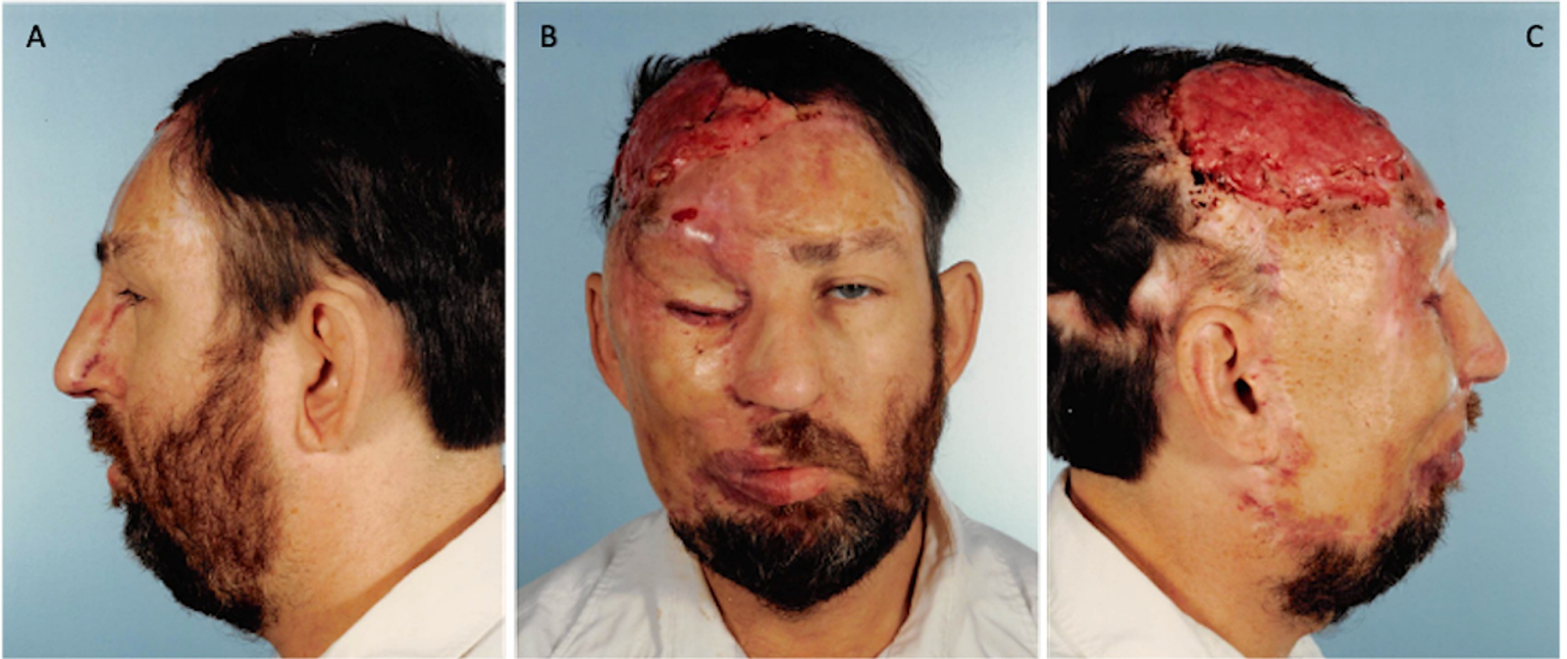
January 15, 1993. Images showing increased granulation tissue of the exposed scalp after debridement and cleaning with Dakin's solution. A= Left profile, B= Frontal, C= Right profile

Approximately 16 months after procedure No. 7, the patient returned to the operating room for multiple revisions including reconstruction of the facial flaps, right lateral and medial canthopexies, and additional skin grafts to the face. One week following surgery, the skin grafts on the scalp, forehead, and portions of the eyebrow and nasolabial area were reportedly removed by the patient. Examination revealed poor hygiene and total loss of the skin graft on the right scalp and forehead, and partial loss of the right eyebrow and nasolabial fold (Figure 8). The wounds were extensively cleaned with Dakin's solution, which was successful in preventing infection and encouraging the formation of granulation tissue (Figure 9). The patient was advised and urged to have additional surgery for an immediate skin graft to the area to avoid infection and malignant transformation of the tissue, but he did not comply.

**Figure 10:**
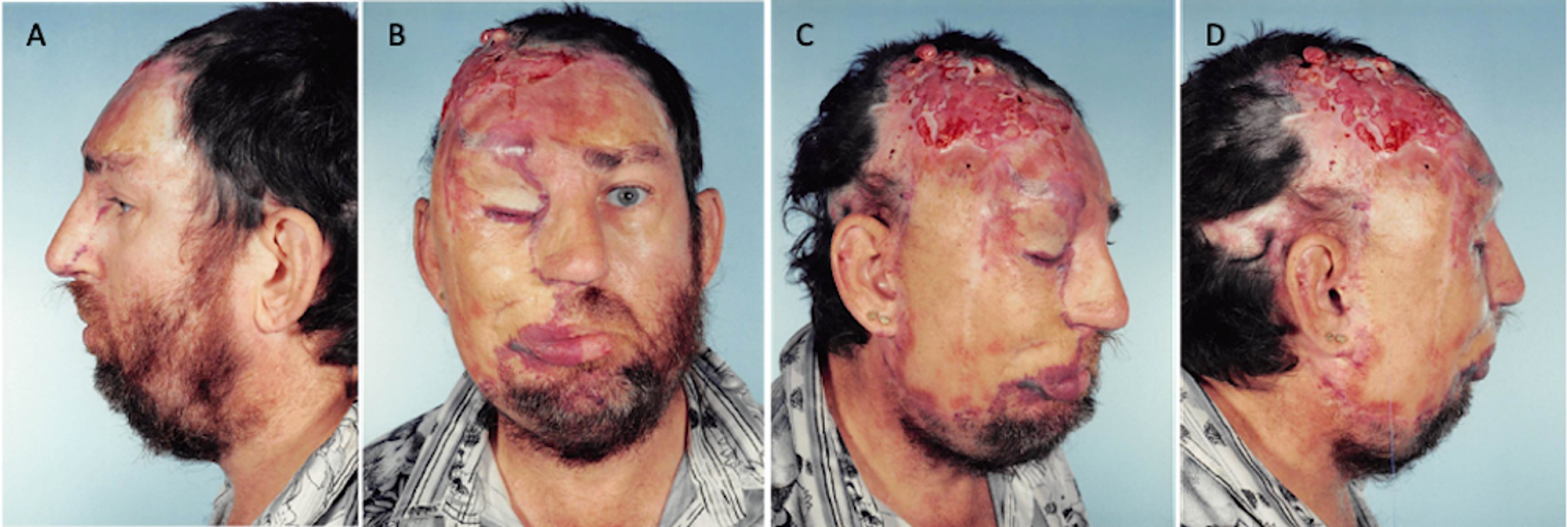
April 24, 1995. Final outcome. A= Left profile, B= Frontal, C= Partial right profile, D= Full right profile

After months of encouragement, the patient agreed to undergo surgery and was admitted to the hospital but ultimately left against medical advice prior to the operation. From 1993 to 1996, the patient continued to follow-up at the office, expressing interest in further surgical intervention but not following through. The last photographic documentation from 1995 portrays the significantly poor outcome following his 10-year treatment (Figure 10).

## Discussion

A major component of Sturge Weber Syndrome is the significant facial disfigurement caused by soft tissue and bony overgrowth secondary to the associated vascular malformation. The surgical treatment of this deformity, however, has received little coverage in the literature [12-15]. Even with full patient cooperation, appropriate surgical intervention by reconstructive surgeons to restore normal anatomy and improve quality of life is extremely challenging and requires multi-staged procedures [16,17]. Patients with Sturge Weber Syndrome may also suffer from significant seizures due to intracranial involvement as well as psychosocial distress related to the disfigurement and resultant social stigmatization [11,18]. This case portrays how this constellation of comorbidities can greatly interfere with post-surgical management and result in poor long-term surgical and aesthetic outcomes.

The current literature recognizes the effect of active mental illness on surgical outcomes, reporting that mental illness is associated with higher rates of postoperative morbidity, additional operations, and financial burden [19,20]. At the time of the initial visit, the patient was regularly visiting a clinical psychologist and was cleared for surgery from a mental health perspective. However, following the difficult surgeries and long duration of complicated postoperative care, the patient became increasingly distressed and began engaging in self-harm including picking and scratching the delicate flaps as well as missing appointments regularly. This resulted in multiple additional unplanned surgeries to address the ensuing defects. The exacerbation of his self-injurious behaviors during the middle stages of treatment led to an inability to fully discontinue treatment due to the acuity of the open wounds and the high risk of worsening complications without intervention. Thus, treatment attempts were continued with increased collaboration with his neurologist and psychologist aimed at controlling his seizure activity and impulsive tendencies. Following multiple attempts to salvage his appearance, the patient became unmotivated and stopped returning to the clinic. 

This case emphasizes the importance of proper and frequent mental health assessments and counseling during the treatment of psychologically distressing conditions, such as facial reconstruction for severe disfigurement secondary to congenital vascular anomalies, which requires long-term compliance and self-motivation from the patient as well as the treating surgeon. Physicians should be aware of the psychosocial distress that accompanies patients with Sturge Weber Syndrome and have the proper means to address such issues.

## Conclusions

The surgical management of facial vascular malformations in Sturge Weber Syndrome is a complicated, multi-stage process that involves both soft tissue and bone reconstruction to restore facial symmetry. Patients with Sturge Weber Syndrome suffer from comorbidities, namely seizures and psychosocial distress related to their condition, which can manifest as self-injury and negatively affect long-term surgical outcomes. Emphasis should be placed on effective psychiatric care and seizure management during the prolonged period of surgical reconstruction.
